# WD repeat domain 5 promotes the development of late-onset preeclampsia by activating nuclear factor kappa B

**DOI:** 10.1590/acb386223

**Published:** 2023-12-01

**Authors:** Xudong Zhao, Fengyun Su, Fanhua Kong, Juan Su, Xiaojing Yang, Lei Li, Aihua Li, Qinwen Li

**Affiliations:** 1 Liaocheng People’s Hospital – Department of Obstetrics and Gynaecology – Liaocheng (Shandong Province) – China.; 2 The Affiliated Taian City Central Hospital of Qingdao University – Taian City Central Hospital – Department of Obstetrics – Taian City (Shandong Province) – China.; 3 The Second Affiliated Hospital of Shandong First Medical University – Second Affiliated Hospital – Department of Pharmacy – Taian City (Shandong Province) – China.; 4 The Affiliated Taian City Central Hospital of Qingdao University – Taian City Central Hospital – Departments of Thoracic Surgery – Taian City (Shandong Province) – China.; 5 The Affiliated Taian City Central Hospital of Qingdao University , Taian City Central Hospital – Department of Obstetrics and Gynecology Color Ultrasound – Taian City (Shandong Province) – China.; 6 Shandong Provincial Hospital Affiliated to Shandong First Medical University – Shandong Provincial Hospital – Department of Obstetrics – Jinan City (Shandong Province) – China.

**Keywords:** Pre-Eclampsia, Trophoblasts, NF-kappa B

## Abstract

**Purpose:**

Over-activation of nuclear factor kappa B (NF-κB) was proven to be involved in the pathogenesis of preeclampsia. However, its regulation mechanism is not clear yet. This paper explored the role of WD repeat domain 5 (WDR5) in the development of late-onset preeclampsia and its relationship with NF-κB.

**Methods:**

WDR5 expression was detected in normal placentas and placentas from late-onset preeclampsia patients. CCK-8 and colony formation assays were conducted to appraise the proliferative ability of trophoblast. Migration and invasion were observed by wound healing and transwell assays. The interaction between WDR5 and NF-κB inhibitor I-kappa-B-alpha (IkBa) was verified by Co-immunoprecipitation analysis. Immunofluorescence was used to analyze the activation of NF-κB. Finally, we tested the role of WDR5 using the mice late-onset preeclampsia model.

**Results:**

WDR5 was highly expressed in the placentas of late-onset preeclampsia patients. WDR5 overexpression suppressed cell proliferation, migration, and invasion in trophoblast. WDR5 could interact with IkBa to activate NF-κB. Knockdown of NF-κB counteracted the anti-proliferative and anti-metastatic effects of WDR5 overexpression in trophoblast. In-vivo studies suggested that targeting WDR5 combated late-onset preeclampsia development.

**Conclusions:**

Our finding provides new insights into the role of WDR5 in late-onset preeclampsia development.

## Introduction

The occurrence of preeclampsia is increasing rapidly, maintaining an upward trend in the last five to 10 years^1^^–^ 3. The incidence of preeclampsia is 3 to 7% worldwide^2^. According to statistics, 10 to 20% of maternal deaths are related to preeclampsia^4^^,^ 5. As we all know, it is critical to establish the placenta and appropriate uteroplacental blood flow for fetal development, maternal well-being, and the physiologic homeostasis of offspring^4^.

During the first trimester of human pregnancy, placental extravillous trophoblasts invade the maternal uterine spiral arteries to replace the vascular smooth muscle and endothelial cells, thus forming high-capacity and low-resistance vessels in the placenta, which is beneficial for the high perfusion of the placenta^6^^–^ 8. Failure of spiral artery remodeling leads to placental ischemia and poor perfusion, a condition that may cause several pregnancy complications, including new-onset maternal hypertension and preeclampsia^7^. According to the gestation age of onset of clinical signs and symptoms, preeclampsia is divided into early-onset (≤ 34 weeks) and late-onset preeclampsia (> 34 weeks)^9^. Although sharing similar maternal features, signs, and symptoms, early-onset and late-onset preeclampsia seem to be originated from different regulation mechanisms. However, the dysfunction of trophoblasts, including decreased proliferation, invasion, and migration, is involved in both subtypes of preeclampsia^10^.

As a key regulator of histone methyltransferase, WDR5 is crucial for histone H3 lysine 4 trimethylation, chromatin remodeling, and transcriptional activation of target genes^11^. It has been well documented that WDR5 is expressed in multiple tumors and participates in tumor vascularization, so as to regulate the proliferation, migration, and invasion of cancer cells^12^. Additionally, a normal level of WDR5 expression is necessary for cell growth and differentiation^13^. The activity of nuclear factor kappa B (NF-κB) was found to be up-regulated in the placentas of preeclampsia patients, and it was proven to be involved in the occurrence and development of preeclampsia^14^. However, the regulation mechanism of NF-κB has not been clarified yet, nor has its relationship with WDR5.

Our previous work has investigated the role of WDR5 in the development of early-onset preeclampsia patients^15^, but it has explored only its role in late-onset preeclampsia. Considering that abnormal proliferation and invasion of trophoblasts are also involved in the progression of late-onset preeclampsia^16^, we proposed a hypothesis that WDR5 was involved in late-onset preeclampsia development by regulating proliferation and invasion, through NF-κB-related signaling. In this study, by utilization of clinical samples in more restricted standards, as well as in-vitro and in-vivo experiments, we investigated the role of WDR5 and NF-κB in late-onset preeclampsia comprehensively.

## Methods

### Tissue collection

From March 1, 2021, to August 1, 2021, placenta tissues from 30 cases of patients with preeclampsia (gestational ages: 36 to 39 weeks) were collected from the Department of Obstetrics and Gynaecology, Liao Cheng People’s Hospital, Department of Obstetrics, Shandong Provincial Hospital Affiliated to Shandong University, and the Department of Obstetrics, Tai’an City Central Hospital. The inclusion criteria of patients were:

- Late-onset preeclampsia diagnosed by medical history and pathology;
- Gestational ages: 36 to 39 weeks;
- Willing to donate placenta tissue for scientific research after delivery.

Meanwhile, placenta tissues from 30 cases of patients at gestational ages of 36 to 39 weeks who received emergency cesarean section due to threatened uterus rupture were also collected as the control group. The gestational ages between the preeclampsia group and the control group were statistically matched. Placenta tissues were taken from the center of the placenta within 30 min after delivery of the placenta. Basic information including age, body mass index (BMI, kg/m^2^), blood pressure (mmHg), and urinary protein (g/24 h) was also gathered.

In this study, patients with diabetes, infectious diseases, circulation system disease, and autoimmune disease were excluded. Besides, patients with intrauterine growth restriction caused by factors other than preeclampsia including underfeeding, intrauterine infection, fetal chromosomal abnormality, uterine malformation, umbilical anomalies, placental abruption, etc. were also excluded.

This study was approved by the Ethics Committee of the Liao Cheng People’s Hospital (approval no. 2018.0407.2), the Shandong Provincial Hospital Affiliated to Shandong University (approval no. 2018-0056), and the Tai’an City Central Hospital (approval no. 2018017v4), and it was conducted in accordance with the Declaration of Helsinki (1964). Informed consent was obtained for experimentation with human subjects.

### Cell culture

The primary human trophoblasts were isolated from the placenta tissues of patients with or without late-onset preeclampsia according to the previously reported method^17^. Briefly, placenta tissues were digested repeatedly with 300 IU/mL deoxyribonuclease (Sigma-Aldrich, St. Louis, MO, United States of America) and 0.25% trypsin (Sigma-Aldrich, St. Louis, MO, United States of America) at 37°C, underwent filtration and discontinuous density gradient centrifugation in a discontinuous Percoll gradient (Sigma-Aldrich, St. Louis, MO, United States of America) to purify the trophoblast cells. Isolated cells were cultured in Dulbecco’s modified eagle medium (high glucose) supplemented with 10% (v/v) fetal bovine serum (FBS) (Gibco, United States of America), 1% penicillin (Gibco, United States of America), and 1% streptomycin (Gibco, United States of America) at 37°C in a 5% CO_2_ atmosphere.

Trophoblast cell line HTR-8/SVneo (ATCC, United States of America) was also cultured in RPMI 1,640 medium supplemented with 10% (v/v) FBS (Gibco, United States of America), 1% penicillin and streptomycin (Gibco, United States of America) at 37°C in a 5% CO^2^ atmosphere.

### Cell transfection

The control siRNA (si-NC), WDR5 siRNA (si-WDR5), IKBa siRNA (si-IKBa), and NF-κB siRNA (si-NF-κB) were generated by GenePharma in Shanghai. The control vector (vector-NC) and WDR5 vector (vector-WDR5) were also generated (GenePharma, Shanghai). Lipofectamine^TM^ 2000 was employed for cell transfection, according to the manufacturer’s instructions.

### Cell counting kit-8 assay

Cells (5,000 cells/well) grown in 96-well plates were cultured for 48 hours, followed by the supplement of CCK-8 solution (Beyotime, China). After 2-4-hour incubation, OD_450nm_ was measured using a microplate reader.

### Colony-formation assay

HTR-8/SVneo cells were seeded into six-well plates at a rate of 300 cells/well and incubated for 10 days. Then, cells were washed twice with PBS and stained with 2% crystal violet solution for 15 min. The number of clones was counted under a microscope.

### Wound healing assay

HTR-8/SVneo cells (1×10^5^) were cultured in 24-well plates up to 90% confluence. Then, cells were scratched with a 10-µL sterile pipette tip. The plate was incubated at 37°C for 24 hours, and the wounded area was photographed before and after 24-hour incubation by a microscope (Leica Microsystems).

### Transwell migration assay

HTR-8/SVneo cells (1×10^5^) were re-suspended in serum-free medium and seeded into the upper compartment of transwell chambers (Corning, NY, United States of America) spreading with 50 µL of Matrigel (Solarbio, Beijing, China). The lower chambers were filled with 600-µL FBS-containing medium. After 24 hours of incubation, the invaded cells were immobilized by 4% paraformaldehyde. Afterwards, 0.1% crystal violet solution was to dye cells for 20 min and photographed under a microscope (Olympus, Tokyo, Japan).

### Real-time quantitative polymerase chain reaction

Total RNA was isolated using TRIzol reagent. Subsequently, RNA was reverse transcribed into cDNA using the prime script reagent RT kit. Then, real-time polymerase chain reactions (PCR) were performed using an ABI Detection System and SYBR Green Premix Ex Taq™. The primer sequences for the genes are as follows: WDR5 sense: 5’-AATATCCGATGTAGCCTGGTC-3’, antisense: 5’-TTGGACTGGGGATTGAAGTTG-3’; PCNA sense: 5’- AAACTAGCTAGACTTTCCTC-3’, antisense: 5’- TCACGCCCATGGCCAGGTTG -3’; Ki-67 sense: 5’- TGCGTTCTGCTCCTACTGCTT-3’, antisense: 5’- CCGTGATCCATTCATTCTGCTTATT-3’; GAPDH sense, 5’-AAAATCAAGTGGGGCGATGCT-3’, antisense: 5’-GGGCAGAGATGATGACCCTTT-3’. The relative expression of the gene was calculated using the 2^−∆∆Ct^ method.

### Western blotting analysis

Total lysates from tissue samples and cell lines were obtained by lysing in RIPA lysis buffer. A bicinchoninic acid assay kit was utilized for the quantification of proteins. Subsequently, SDS-PAGE was used to segregate protein samples, followed by transfer to polyvinylidene difluoride (PVDF) membrane. Specific antibodies against WDR5, PCNA (Proliferating Cell Nuclear Antigen), Ki67, matrix metalloproteinase (MMP)2, MMP9, IkBa, and glyceraldehyde 3-phosphate dehydrogenase (GAPDH) were incubated with membranes overnight at 4°C. Protein bands were detected with enhanced chemiluminescence (ECL) and analyzed using ImageJ software.

### Immunohistochemistry

Immunohistochemistry (IHC) was performed in paraffin-embedded sections using an IHC kit (SV0002, BOSTER, China). Briefly, after being deparaffinized and rehydrated, the sections were blocked using 5% bovine serum albumin for 30 min, and then incubated with antibodies anti-WDR5 (ab178410, Abcam, United States of America) or anti-NF-κB p65 (ab32536, Abcam, United States of America) overnight at 4°C, followed by being incubated with the secondary antibody provided in the kits for another 30 min. The section was then stained with 3,3’-diaminobenzidine, and it was observed and photographed using an Olympus microscopy. Protein expression was quantified according to the H-score, which was calculated using the following formula: H-score ¼ Pi (i), where i is the intensity of staining with a value of 1, 2, or 3 (weak, moderate, or strong, respectively), and Pi is the percentage of stained cells for each intensity in the range of 0–100%^18^.

### Immunofluorescence

Cells were fixed with 4% formaldehyde and incubated overnight at 4°C with primary antibodies anti-tubulin (ab7291, Abcam, United States of America) or anti-NF-κB p65 (ab32536, Abcam, United States of America). Cells were then incubated with the goat anti-rabbit secondary antibody (BA1031 and BA1032, BOSTER, China) at 37°C for another 30 min. The nucleus was strained by 4’,6-diamidino-2-phenylindole (DAPI) (Beyotime, China). Cells were observed by laser scanning confocal microscope (Leica, Germany), and results were analyzed by Leica Application Suite X (Leica, Germany).

### Co-immunoprecipitation

Cells were lysed in immunoprecipitation (IP) lysis buffer (Beyotime, Shanghai, China) with a cocktail of protease/phosphatase inhibitors (Beyotime, Shanghai, China). Then, total proteins were incubated with anti-WDR5 or anti-IgG overnight at 4°C. Subsequently, protein A/Gcoated magnetic beads were used to capture protein complexes at 4°C for 6 hours. The immunoprecipitated proteins were examined by western blotting analysis after being washed in lysis buffer for 3 minutes.

### Animal model development

All animal experiments comply with the ARRIVE guidelines and were carried out in accordance with the U.K. Animals (Scientific Procedures) Act, 1986, and the National Institutes of Health guide for the care and use of laboratory animals (NIH Publications No. 8023, revised 1978).

In this study, ICR (Institute of Cancer Research) female mice (n = 24, 200–250 g) were purchased from the Animal Experiment Center of Shandong University School of Medicine (experimental animal quality certificate no. 42007800001156). Animal experiments were approved by the Medical Ethics Committee of Shandong University School of Medicine, and the process was carried out in strict accordance with the regulations for the management of experimental animals.

Mice were fed adaptively in separate cages for four or five days in the animal room at a room temperature of 20–25°C, with free food and water, and the bedding in the rearing cage was changed every three or four days. The female mice were marked with picric acid, and, when the female mice were in estrus, they were housed in cages with male mice at a ratio of 2:1 after 8 p.m. Female mice were given vaginal smears before 8 a.m. the next morning. When sperm was found, it was considered to be the first day of pregnancy, and they were kept in separate cages.

Pregnant mice were randomly divided into three groups with eight mice in each group. The first group was a normal pregnancy group, and the latter two groups were injected with L-NG-nitro arginine methyl ester (L-NAME) on the 16^th^ day of gestation for the modeling of late-onset preeclampsia^19^. From the 16^th^ day of gestation, L-NAME solution (125 mg/kg/d),si-NC (20 M/d) and/or si-WDR5 (20M/d) were subcutaneously injected. The injection was performed until the 19^th^ day of pregnancy, for a total of four days.

The blood pressure of the mice was measured with a non-invasive mouse tail artery manometer before pregnancy and every other morning from the second day of pregnancy, and the measurement was repeated three to five times each time. The mice were placed in metabolic cages on the 20^th^ day of gestation, without food or water. The 24-hour urine of the mice was collected from 10 a.m. to 10 a.m. the next day, the collected urine was centrifuged at 2,000 rpm for 5 minutes, and the urine protein content was detected by the BCA method.

### Statistical analysis

Data from three sets of independent experiments are displayed as means ± standard deviation (SD). Differences among diverse groups were confirmed using one-way analysis of variance (ANOVA), and differences were determined to be statistically significant when *P*<0.05.

## Results

### WDR5 was increased in placentas of late-onset preeclampsia patients, and its level was positively correlated with the severity of patients

**Figure 1:**
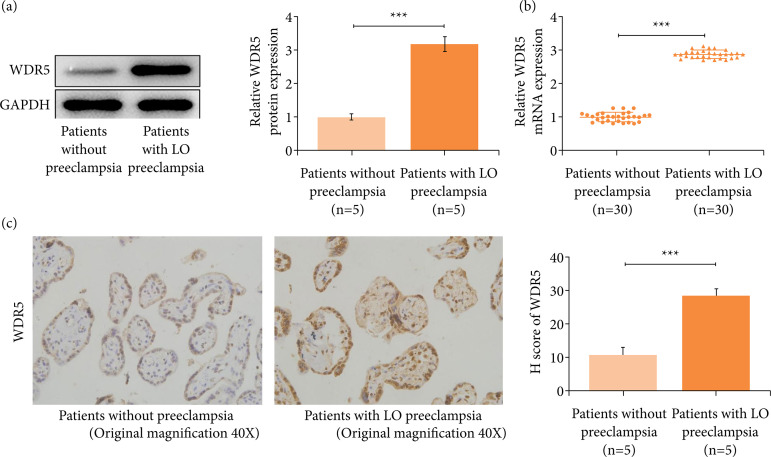
WDR5 is increased in placentas of the LO preeclampsia patients. **(a)** The WDR5 protein level was upregulated in placentas of the LO preeclampsia patients compared to that of patients without preeclampsia. **(b)** mRNA of WDR5 was upregulated in placentas of the LO preeclampsia patients compared to that from patients without preeclampsia. **(c)** Results of the immunohistochemistry assay verified the increase of WDR5 in placentas of the LO preeclampsia patients.

WDR5: WD repeat domain 5; GAPDH: glyceraldehyde 3-phosphate dehydrogenase: LO: late-onset; ***P < 0.001.

We first analyzed the WDR5 expression in placentas. Results showed that the protein and mRNA levels of WDR5 were visibly increased in placentas of late-onset preeclampsia patients (Figs. 1a–1c). Besides, we found that the mRNA level of WDR5 in placentas of late-onset preeclampsia patients had a positive relationship with the severity of this disease: highest expression of WDR5 was observed in patients who had higher blood pressure, patients with gestation less than 37 weeks, patients who had a higher concentration of urinary protein, patients with low-birth-weight infants, and patients with lower weight of placentas (Table 1).

**Table 1:** The relationship between WDR5 expression in placentas from preeclampsia patients and patients’ clinical characteristics.

Characteristics	Case (n = 30)	WDR5 mRNA level	*t/F*	P-value
**Maternal age (year old)**			**-1.717**	**0.097**
< 35	20	0.994 ± 0.025		
≥ 35	10	1.012 ± 0.031		
**Body mass index (kg/m^2^)**			**0.896**	**0.409**
< 18.5	7	0.988 ± 0.017		
18.5–24.9	16	1.013 ± 0.019		
≥ 25	7	0.982 ± 0.021		
**Gestational age (week)**			**16.471**	**< 0.001**
< 37	8	1.067 ± 0.034		
≥ 37	22	0.815 ± 0.045		
**Systolic pressure (mmHg)**			**-22.258**	**< 0.001**
< 160	23	0.806 ± 0.019		
≥ 160	7	1.059 ± 0.028		
**Diastolic pressure (mmHg)**			**-13.284**	**< 0.001**
< 110	24	0.821 ± 0.041		
≥ 110	6	1.045 ± 0.036		
**Proteinuria (g/24 h)**			**-12.448**	**< 0.001**
< 5	19	0.824 ± 0.078		
≥ 5	11	1.102 ± 0.045		
**Neonatal weight (kg)**			**10.117**	**< 0.001**
< 2.5	7	1.034 ± 0.057		
≥ 2.5	23	0.811 ± 0.016		
**Placental weight (g)**			**28.611**	**< 0.001**
< 500	12	1.123 ± 0.036		
≥ 500	18	0.815 ± 0.011		

WDR5: WD repeat domain 5.

### WDR5 overexpression abrogated cell proliferation and clonal growth in trophoblasts

**Figure 2:**
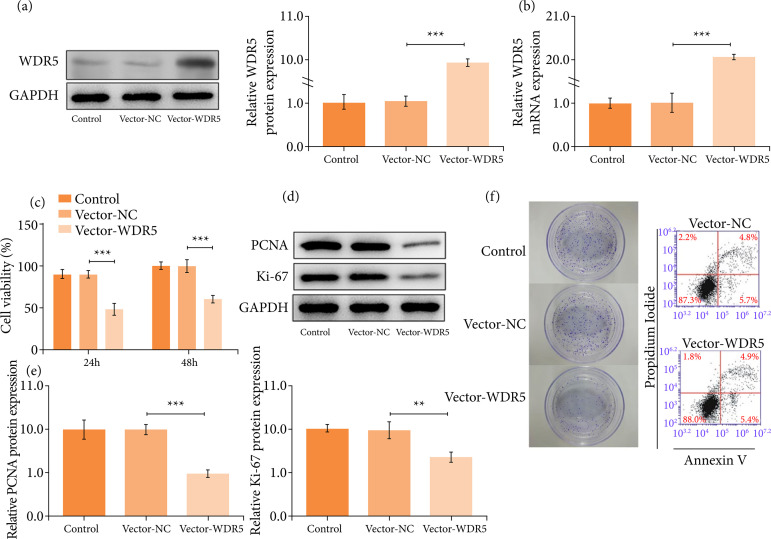
WDR5 overexpression abrogated cell proliferation and clonal growth in trophoblast cells. (a and b) WDR5 expression was measured by western blotting analysis and real-time quantitative polymerase chain reaction. **(c)** The proliferation of trophoblast cells was measured by a CCK-8 assay. (d and e) The protein levels of PCNA and Ki67 were detected by western blotting analysis. **(f)** The clonal growth of trophoblast cells was detected by colony formation assay.

WDR5: WD repeat domain 5; GAPDH: glyceraldehyde 3-phosphate dehydrogenase; NC: negative control; **P < 0.01; ***P < 0.001.

To further verify the role of WDR5, we overexpressed WDR5 in trophoblasts (Figs. 2a and 2b). We observed that WDR5 overexpression impeded the proliferation of trophoblast cells (Fig. 2c) and distinctly depleted the protein levels of proliferation markers including PCNA and Ki67 (Figs. 2d and 2e). Meanwhile, the upregulation of WDR5 hampered the clonal growth of trophoblast cell (Fig. 2f). No significant difference was observed in the apoptosis rates between trophoblasts with or without WDR5 overexpression ((Fig. 2g). These findings implied that WDR5 overexpression prominently attenuated cell proliferation and clonal growth in trophoblast cells.

### WDR5 overexpression weakened the migratory and invasive capacities of trophoblasts

**Figure 3:**
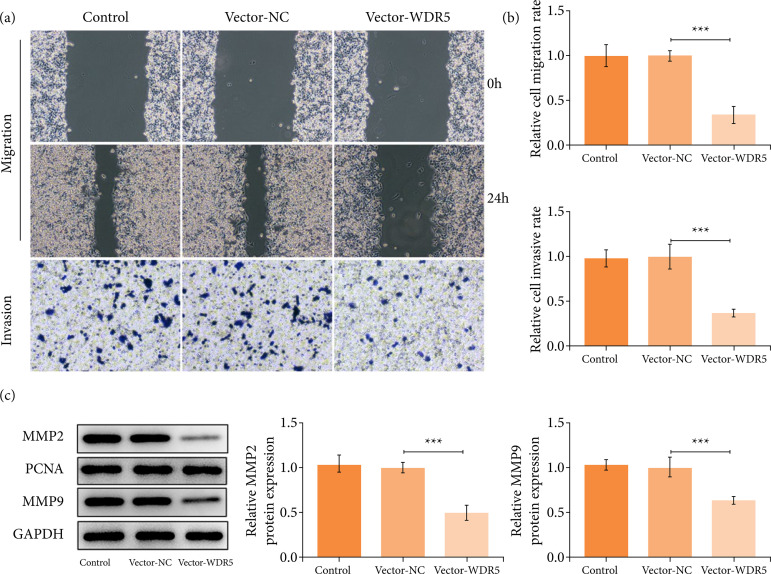
WDR5 overexpression suppressed the migration and invasive abilities of trophoblast cells. (a and b) Wound healing assay and transwell assay respectively appraised cell migration and invasion. **(c)** Western blotting analyzed MMP2 and MMP9 expression at protein levels.

WDR5: WD repeat domain 5; GAPDH: glyceraldehyde 3-phosphate dehydrogenase; NC: negative control; MMP: matrix metalloproteinase; **P < 0.01; ***P < 0.001.

We next tested the effect of WDR5 overexpression on the migratory and invasive capacities of trophoblasts. Results showed that the upregulation of WDR5 significantly arrested the migration and invasion of HTR-8/SVneo cells (Figs. 3a and 3b). Moreover, the protein levels of key factors regarding invasion including MMP2 and MMP9 were also reduced significantly in trophoblast cells upon WDR5 overexpression (Fig. 3c).

### WDR5 activated NF-κB by binding to IkBa

**Figure 4:**
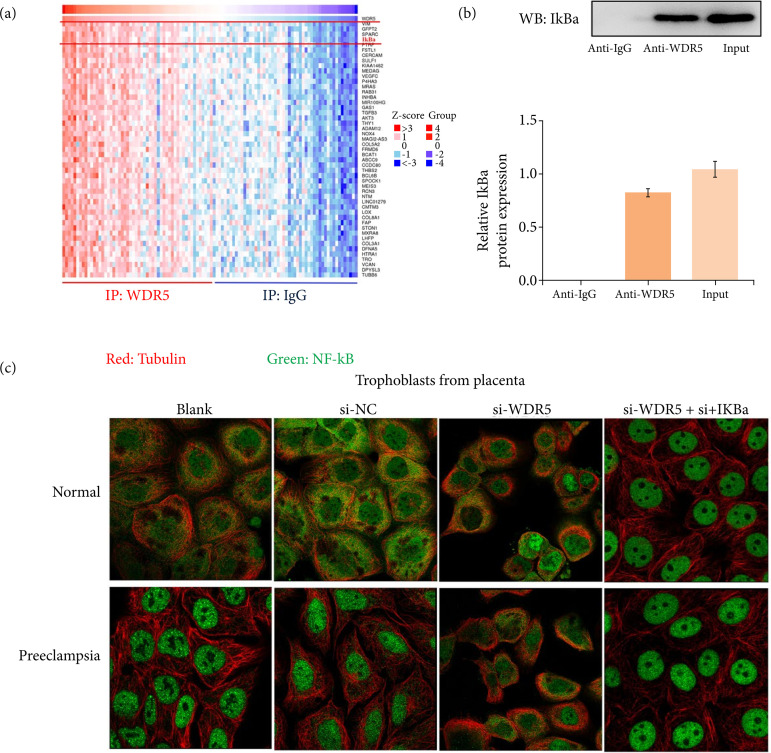
WDR5 activated NF-κB by binding to IkBa. **(a)** The top 50 proteins interacting with WDR5 in the heat map (red: proteins positively correlated; blue: proteins negatively correlated). **(b)** Co-IP assays verified the interaction between WDR5 and IkBa. **(c)** Immunofluorescence estimated the activation of NF-κB in trophoblast cells from placental tissues.

WDR5: WD repeat domain 5; WB: western blotting; IP: immunoprecipitation; NF-κB: nuclear factor kappa B; **P < 0.01; ***P < 0.001.

To dissect the underlying mechanism, we next used the HitPredict database to predict the interacting proteins with WDR5. The top 50 proteins with potential interaction with WDR5 were presented in the heat map, and IkBa showed a strong possibility to interact with WDR5 (Fig. 4a). Co-IP analysis validated the interaction between WDR5 and IkBa (Fig. 4b). The location of NF-κB reflects its activity, with nuclear protein indicating the active form, while cytoplasmic protein indicates the inactive form. We next evaluated the activity of NF-κB using immunofluorescence. As we expected, compared to normal placenta, trophoblasts from placenta of late-onset preeclampsia patients showed over-activated NF-κB (Fig. 4c), while the knockdown of WDR5 rescued phenomenon of over-activated NF-κB observed in trophoblasts from placenta of late-onset preeclampsia patients (Fig. 4c). Additionally, using si-IKBa to silence IKBa eliminated the function of si-WDR5 (Fig. 4c). These results indicate that WDR5 activated NF-κB through IkBa.

### Gain of WDR5 suppressed proliferative ability of trophoblasts through NF-κB

**Figure 5:**
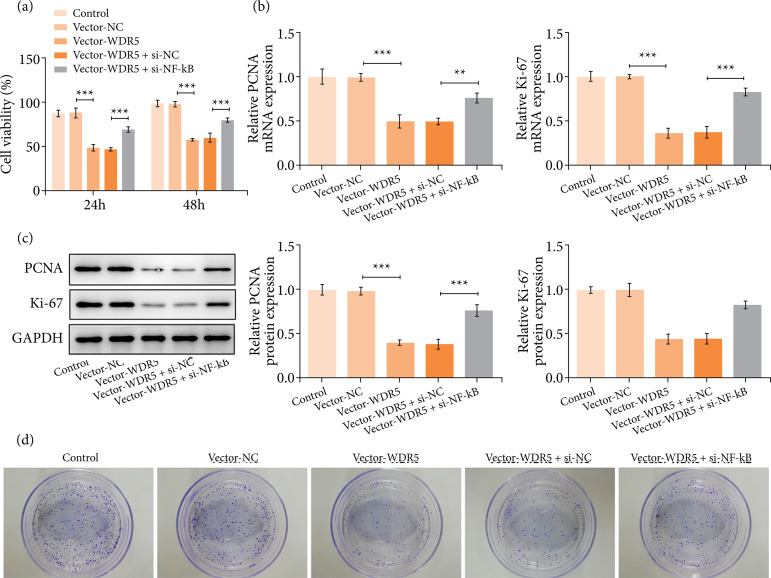
WDR5 overexpression suppressed the proliferation and clonal growth of trophoblast cells through NF-κB. **(a)** The proliferation of trophoblast cells was measured by a CCK-8 assay. (b and c) The expressions of PCNA and Ki-67 at the mRNA level and protein level were measured by real-time quantitative polymerase chain reaction and western blotting analysis, respectively. **(d)** The clonal growth of trophoblast cells was detected by colony formation assay.

WDR5: WD repeat domain 5; NC: negative control; NF-κB: nuclear factor kappa B; GAPDH: glyceraldehyde 3-phosphate dehydrogenase; **P < 0.01; ***P < 0.001.

HTR-8/SVneo cells were transfected with vectors or siRNAs to upregulate the expression of WDR5 or silence NF-κB. Results revealed that the suppressed HTR-8/SVneo cell proliferation caused by WDR5 overexpression was recused upon the knockdown of NF-κB (Fig. 5a). Additionally, enhanced PCNA and Ki-67 levels after the co-transfection of vector-WDR5 and si-NF-κB validated the mentioned findings (Figs. 5b and 5c). Furthermore, silencing NF-κB abrogated the suppression effect of WDR5 overexpression on the clone-forming ability of HTR-8/SVneo cells (Fig. 5d).

### Gain of WDR5 hindered trophoblast cell migration and invasion through NF-κB

**Figure 6:**
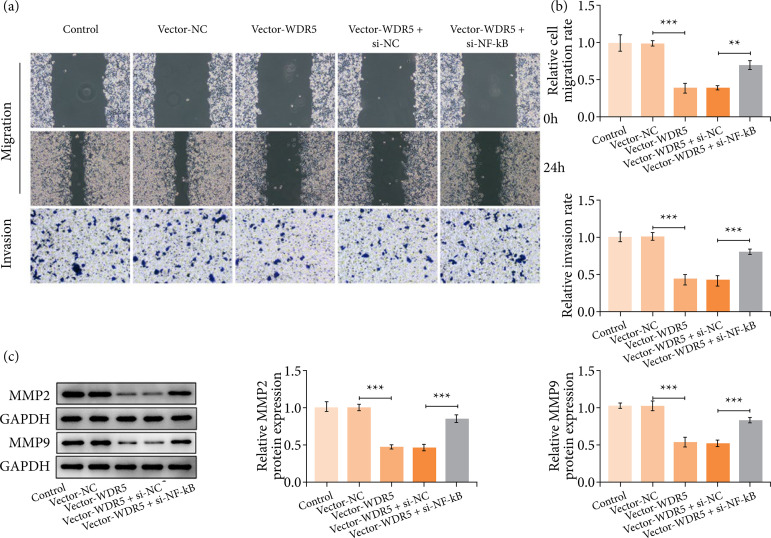
WDR5 overexpression hindered trophoblast cell migration and invasion through NF-κB. (a and b) Wound healing assay and transwell assay respectively appraised cell migration and invasion. **(c)** Western blotting analyzed MMP2 and MMP9 protein levels.

WDR5: WD repeat domain 5; NC: negative control; NF-κB: nuclear factor kappa B; MMP: matrix metalloproteinase; GAPDH: glyceraldehyde 3-phosphate dehydrogenase; **P < 0.01; ***P < 0.001.

Also, it was revealed that inhibiting NF-κB expression in WDR5-overexpressing HTR-8/SVneo cells rescued the cellular migration and invasive abilities (Figs. 6a and 6b). In addition, the increased protein levels of MMP2 and MMP9 were also observed (Fig. 6c).

### Silencing of WDR5 alleviated late-onset preeclampsia in mouse model

**Figure 7:**
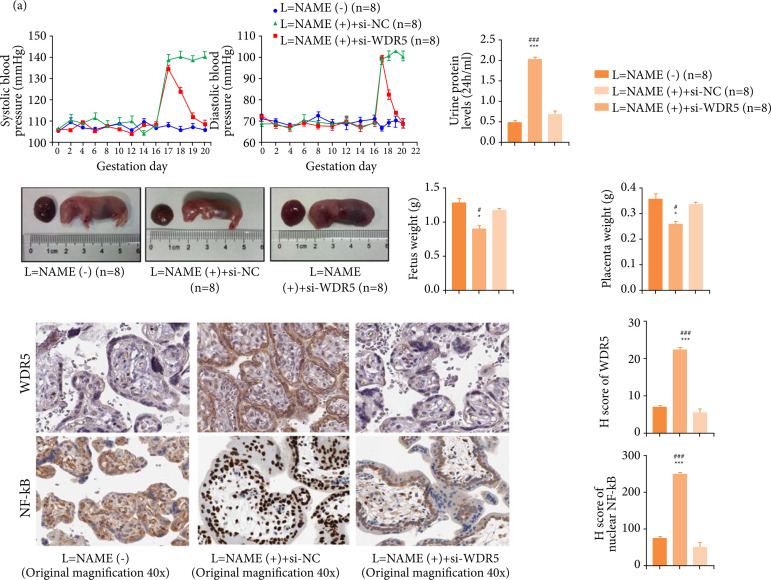
Silencing WDR5 inhibited the late-onset preeclampsia development in mice. **(a)** Effects of si-WDR5 on blood pressure in late-onset preeclampsia mice. **(b)** Effects of si-WDR5 on urinary protein in late-onset preeclampsia mice. **(c)** Effects of si-WDR5 on offspring development of late-onset preeclampsia mice. **(d)** WDR5 expression level and nuclear NF-κB level were significantly higher in the late-onset preeclampsia group than that in the normal group and the si-WDR5 group.

WDR5: WD repeat domain 5; NC: xxxx; NF-κB: nuclear factor kappa B; *P < 0.05, compared with the L-NAME (-) group; ***P < 0.001, compared with the L-NAME (-) group; #*P* < 0.05, compared with the L-NAME (+) + si-WDR5 group; ###*P* < 0.001, compared with the L-NAME (+) + si-WDR5 group.

We then tested the therapeutic value of WDR5 in late-onset preeclampsia using the mouse model. The blood pressure of the mice in the normal pregnancy group was stable throughout the pregnancy. In late-onset preeclampsia group, the blood pressure after injection of the modeling agent L-NAME increased significantly (*P*<0.01, Fig. 7a). The blood pressure of mice in the L-NAME + si-WDR5 group increased, but it dropped back to the normal level quickly (*P* > 0.05, Fig. 7a). The 24-hour urine of the three groups was collected on the 20^th^ day of gestation in mice. Results showed that the 24-hour urine protein level of the late-onset preeclampsia group was significantly higher than that of the normal group and si-WDR5 group (*P* < 0.001, Fig. 7b).

The mice underwent cesarean section on the 21^st^ day of gestation, and the weights of the fetus and the placentas were recorded. Compared with the normal pregnancy group and the si-WDR5 group, the weights of the fetus and the placenta in the late-onset preeclampsia group decreased significantly (*P* < 0.05). There was no significant difference in the weight of offspring and placenta between the normal group and the si-WDR5 group (*P* > 0.05, Fig. 7c). Moreover, WDR5 expression level and nuclear NF-κB level were significantly higher in the late-onset preeclampsia group than that in the normal group and the si-WDR5 group (*P* < 0.001, Fig. 7d). These results suggested that targeting WDR5 could suppress the progression of late-onset preeclampsia^20^^,^ 21.

## Discussion

In the current study, for the first time, we investigated the function of WDR5 in the development of late-onset preeclampsia by both in-vitro and in-vivo experiments, and results indicate that WDR5 promotes the development of late-onset preeclampsia by interacting with IkBa and thus activating NF-κB. Overexpression of WDR5 leads to the attenuated proliferation and invasion of trophoblasts. Besides, we found that the expression level of WDR5 in placenta tissues is positively associated with the severity of preeclampsia, indicating that WDR5 may serve as a biomarker to monitor the progress of late-onset preeclampsia, and to predict the prognosis of this disease.

As a key regulator of histone methyltransferase, WDR5 is crucial for histone H3 lysine 4 trimethylation, chromatin remodeling, and transcriptional activation of target genes^11^. Our previous study has proved that WDR5 is involved in the development of early-onset preeclampsia^15^. Notably, the expression patterns of WDR5 between early-onset preeclampsia and late-onset preeclampsia are not the same^15^, and its role in late-onset preeclampsia is rarely reported. In this study, for the first time, we prove that the increased WDR5 in the placentas results in the attenuated proliferation and invasion of trophoblasts, while targeting WDR5 may serve as an approach for the treatment of late-onset preeclampsia.

To further dissect the molecular mechanism of WDR5 functions, we tried to find the protein which interacts with WDR5. Results indicate that IkBa, an endogenous inhibitor of NF-κB, can bind to WDR5. In mammals, IkBa inhibits the activation of NF-κB by binding to NF-κB and preventing it from forming dimer and entering the nucleus, and thus NF-κB is detained in the cytoplasm as the inactive form^22^. Normally, expressed NF-κB is a key mediator for placental formation^23^. Since insemination, it stimulates the production of proinflammatory cytokines in uterine epithelial cells, leading to the activation of macrophages, uterine natural killer cells, and other leukocytes^24^. Trophoblast-macrophage crosstalk is critical for engraftment and spiral artery remodeling^25^. NF-κB regulates this process by altering cytokine expression, as well as cellular phenotype and function. However, results of a series of studies have proven that over-activated NF-κB affects trophoblasts functions through a variety of mechanisms^26^^–^ 29.

During preeclampsia, the placental syncytiotrophoblast stress is induced by excessive inflammation and increased NF-κB activation^30^. Placental syncytiotrophoblast stress has been found to play a crucial role in late-onset preeclampsia progression, and it is closely related to the dysfunction of trophoblast^31^. A study by Sha et al. indicated that selective inhibition of NF-κB or the NF-κB activation pathway reduced the symptoms of preeclampsia in a rat model^28^.

In our study, we found that WDR5 weakened the proliferation and invasion of trophoblasts and promoted the development of late-onset preeclampsia by activating NF-κB. Our study unmasked that NF-κB over-activation was gained in trophoblasts to function as a regulator to suppress proliferation and invasion in trophoblasts. Moreover, we proved that targeting WDR5 alleviated late-onset preeclampsia progression significantly in mice model, indicating that WDR5 has therapeutic potential in late-onset preeclampsia.

## Conclusion

For the first time, this study found that increased WDR5 in the placenta is involved in the development of late-onset preeclampsia. Mechanically, WDR5 binds to IkBa to activate NF-κB, and further impairs trophoblast cells proliferation, invasion, and migration. These results will help us to understand the pathogenesis of late-onset preeclampsia better and are also conducive to the discovery of new therapeutic targets for late-onset preeclampsia.

## Data Availability

The data will be available upon request.
